# Characteristics of primary care and rates of pediatric hospitalizations in Brazil

**DOI:** 10.11606/s1518-8787.2020054001784

**Published:** 2020-03-25

**Authors:** Lívia Anniele Sousa Lisboa, Rejane Christine de Sousa Queiroz, Erika Bárbara Abreu Fonseca Thomaz, Núbia Cristina da Silva, Thiago Augusto Hernandes Rocha, João Ricardo Nickenig Vissoci, Catherine Ann Staton, Adriana Lein, Vanda Maria Ferreira Simões, Elaine Thumé, Luiz Augusto Facchini

**Affiliations:** I Universidade Federal do Maranhão Programa de Pos graduação em Saúde Pública São LuísMaranhão Brasil Universidade Federal do Maranhão. Programa de Pos graduação em Saúde Pública. São Luís, Maranhão, Brasil.; II Universidade Federal do Maranhão Programa de Pos graduação em Saúde Pública Departamento de Saúde Pública São LuísMaranhão Brasil Universidade Federal do Maranhão. Programa de Pos graduação em Saúde Pública. Departamento de Saúde Pública. São Luís, Maranhão, Brasil.; III Universidade Federal de Minas Gerais Observatório de Recursos Humanos em Saúde Belo HorizonteMinas Gerais Brasil Universidade Federal de Minas Gerais. Observatório de Recursos Humanos em Saúde. Belo Horizonte, Minas Gerais, Brasil.; IV Organização Pan Americana de Saúde BrasíliaDistrito Federal Brasil Organização Pan Americana de Saúde: OPAS/WHO - Brasília, Distrito Federal, Brasil.; V Duke University Duke Global Health Institute DurhamNorth Carolina U.S Duke University. Duke Global Health Institute. Durham, North Carolina. U.S.; VI Universidade Federal de Pelotas Programa de Pós-graduação em Enfermagem Departmento de Enfermagem PelotasRio Grande do Sul Brasil Universidade Federal de Pelotas. Programa de Pós-graduação em Enfermagem. Departmento de Enfermagem. Pelotas, Rio Grande do Sul, Brasil.; VII Universidade Federal de Pelotas Programas de Pós-graduação em Epidemiologia e Enfermagem Departmento de Medicina Social PelotasRio Grande do Sul Brasil Universidade Federal de Pelotas. Programas de Pós-graduação em Epidemiologia e Enfermagem. Departmento de Medicina Social. Pelotas, Rio Grande do Sul, Brasil.

**Keywords:** Primary Health Care, Health Care Quality, Access, and Evaluation, Patient Admission, Ecological Studies

## Abstract

**OBJECTIVE:**

To evaluate the association among characteristics of primary health care center (PHCC) with hospitalizations for primary care sensitive conditions (PCSC) in Brazil.

**METHOD:**

In this study, a cross-sectional ecological study was performed. This study analyzed the 27 capitals of Brazil’s federative units. Data were aggregated from the following open access databases: National Program for Access and Quality Improvement in Primary Care, the Hospital Information System of Brazilian Unified Health System and Annual Population Census conducted by the Brazilian Institute of Geography and Statistics. Associations were estimated among characteristics of primary care with the number of three PCSC as the leading causes of hospitalization in children under-5 population in Brazil: asthma, diarrhea, and pneumonia.

**RESULTS:**

In general, PHCC showed limited structural adequacy (37.3%) for pediatric care in Brazil. The capitals in South and Southeast regions had the best structure whereas the North and Northeast had the worst. Fewer PCSC hospitalizations were significantly associated with PHCC which presented appropriate equipment (RR: 0.98; 95%CI: 0.97–0.99), structural conditions (RR: 0.98; 95%CI: 0.97–0.99), and signage/identification of professionals and facilities (RR: 0.98; 95%CI: 0.97–0.99). Higher PCSC hospitalizations were significantly associated with PHCC with more physicians (RR: 1.23, 95%CI: 1.02–1.48), it forms (RR: 1.01, 95%CI: 1.01–1.02), and more medications (RR: 1.02, 95%CI: 1.01–1.03)

**CONCLUSION:**

Infrastructural adequacy of PHCC was associated with less PCSC hospitalizations, while availability medical professional and medications were associated with higher PCSC hospitalizations.

## INTRODUCTION

In 2017, an estimated amount of 5.4 million children under 5 years old died. More than half of these children deaths occur because of conditions that could be prevented or treated with access to simple, affordable interventions^1^. The World Health Organization estimates that approximately half of these deaths are avoidable by appropriate preventative services^2^. Primary care sensitive conditions (PCSC) are considered avoidable with adequate and timely interventions at the primary care level^3^. Targeting PCSC as preventive actions is particularly urgent in low- and middle-income countries (LMIC), where a disproportionate amount of children under-5 mortality occurs (nearly 99% for infants); concurrent with higher rates of poverty, other complexities in LMIC include the quality of primary care structural (e.g. lack of clean water and sanitation) and organizational (e.g. governance challenges, and under-resourced health systems) characteristics^1,4^_._

In Brazil, an upper-middle income country with a universal health system focused heavily on primary care^5^, studies have found that at least 44.1% of children under-5-hospitalizations were due to PCSC, compared with an estimated 27% for total hospitalizations^6-8^. At the national level, Brazil has a current rate (2016) of under-5 mortality of 16/1,000 live births, meeting the United Nations Sustainable Development Goal of fewer than 25/1,000 live births by 2030^9,10^. However, regional differences in disease burden within Brazil, largely caused by socioeconomic inequality, result in disparities in under-5 mortality^11^. Then greater research at national-level is necessary to identify and understand the primary care predictors of hospitalizations for PCSC specific to pediatric populations across regions.

In a preliminary study we found, in the Brazilian municipalities, that basic health structure affected the domain of the hospitalizations by specific conditions of primary attention^12^. Existing literature on predictors of hospitalizations for PCSC from Brazil tends to focus on noncommunicable diseases among adult populations^13,14^.

In 2012, the Brazilian Ministry of Health undertook the first nationwide diagnostic census of health centers, the National Program for Access and Quality Improvement in Primary Care. This program was designed as an external evaluation of health centers infrastructure regarding aspects such as structure and process involved in service delivery^15^. Using these data, the relationship between hospitalizations for PCSC and characteristics of primary care in Brazil has been analyzed by different approaches. Studies have found negative correlations between hospitalizations for PCSC and Family Health Strategy (FHS) coverage^16^, primary care financing^17^, performances of health care providers, and access to health facilities^18^. Notwithstanding, an evaluation of the current literature reveals a lack of studies addressing avoidable hospitalizations in pediatric populations that account for predictors related to characteristics and their relationship to ecological-level socioeconomic and health care delivery factors.

Avoidable under-5-hospitalizations in Brazil result from the complex interplay of multilevel predictors. This study aims to measure and to describe the relationship between characteristics of primary health care centers (PHCC) in Brazilian capital cities and rates of under-5 hospitalizations for PCSC.

## METHODS

### Study Design and Ethical Approval

This is an ecological observational study based on secondary data. The capital cities of Brazil’s federative units (26 states and the federal district of Brasília) are the unit under analysis. In Brazil, administration of primary care is decentralized among states and cities that are divided into macro- and micro-regions for planning processes^19^. Considering this organization, cities are an appropriate unit of analysis, as both infrastructure and resources of the health system depend on the cities political economies, and they have been shown to vary significantly across cities even within the same state or region^20^. This study was carried out in accordance with the Strengthening the Reporting of Observational studies in Epidemiology (STROBE) Statement guidelines for observational studies. Ethical approval was granted by the Research Ethics Committee of Pelotas University in May, 2012.

### Data Sources

Data were collected from 2012, when the corresponding aggregated population of all federative unit capitals totaled 45,852,569 inhabitants with an approximate 47% of coverage by the Family Health Strategy, the Brazilian central policy for primary care^21^.

#### PCSC indicators

The number of hospitalizations was the dependent variable due to the three most common causes of under-5 PCSC hospitalizations in Brazil (asthma, pneumonia and diarrhea/gastroenteritis). Data regarding under-5 PCSC hospitalizations from January–December 2012 were obtained online from the Hospital Information System of the Unified Health System in SUS Department of Informatics (SIH-DATASUS), which maintains records of public hospitalizations, including information about cause, location, and patient demographics. The three main causes of hospitalizations were selected for the corresponding patients by the International Classification of Diseases — ICD-10, asthma (J45), bacterial pneumonia (J13, J14, J15.3, J15.4, J15.8, J15.9 and J18.1) and presumed infectious gastroenteritis (A08)^22^ (Figure).

**Figure:**
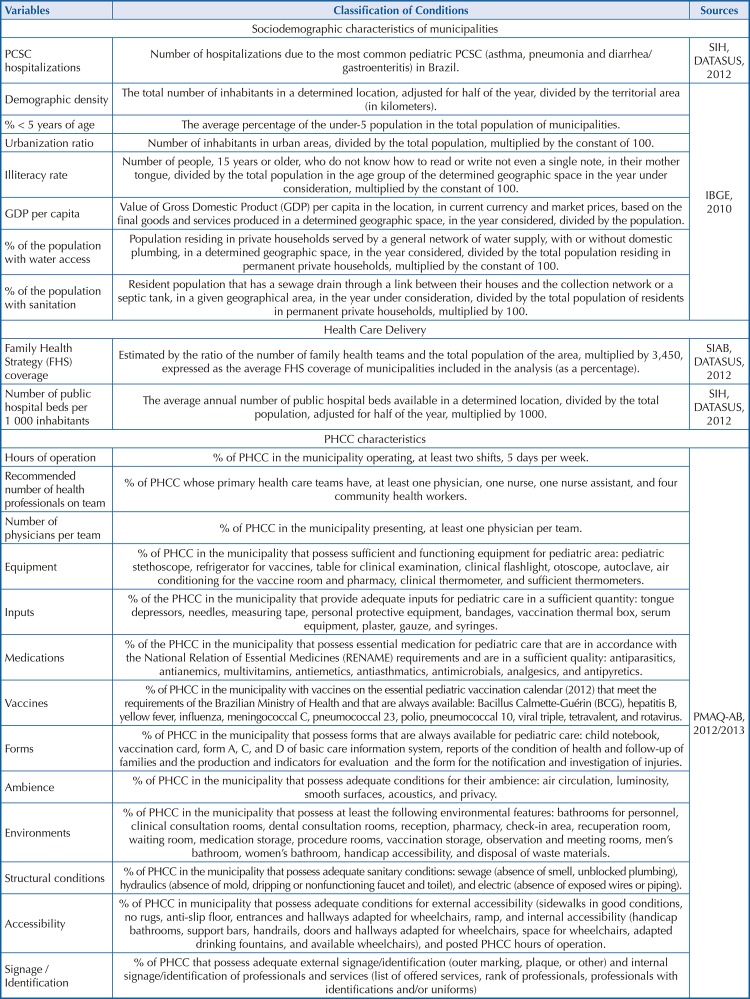
Variable Descriptions. Brazil. 2012–2013.

#### Sociodemographic characteristics

Socioeconomic variables were obtained from a public database of the Brazilian census (Brazilian Institute for Geography and Statistics — IBGE). Specific variables were: demographic density, population of children under-5 years old, urbanization ratio (number of inhabitants in urban areas, divided by the total population, multiplied by the constant of 100), illiteracy rate, Gross Domestic Product (GDP) per capita, percentage of the population with water access, and percentage of the population with sanitation.

#### Health care delivery

Health care delivery variables were extracted from the online Basic Attention Information System of the Unified Health System Department of Informatics. Indicators were the Family Health Strategy (FHS) coverage (proportion of population covered by health teams that work with the FHS assistance model) and the number of public hospital beds per 1000 inhabitants (Figure).

#### PHCC characteristics

PHCC variables were obtained from the database of the National Program for Access and Quality Improvement in Primary Care (PMAQ-AB) conducted between 2012 and 2013^15^. The PMAQ-AB evaluation (Module 1) performed a census of the primary care centers in Brazil. One member of the primary care team at each primary care center answered this census, by an electronic questionnaire. Further information on the PMAQ-AB can be found elsewhere^15^. Characteristics of the PHCC structure were assessed in 13 dimensions grouped into: hours of operation, recommended number of health care professionals, number of physicians per team, equipment, inputs, medications, vaccines, forms, ambience, environments, structural conditions, accessibility, and signage/identification (Figure).

This classification was based on the Manual of Physical Structure of the Basic Units of Health: Family Health^23^, the External Instructive Manual of the National Program for Access and Quality Improvement for Primary Care teams^15^, primary care indicators^16^ and consultations with specialists. A “general structure” variable was generated combining all 13 dimensions. Each of the 13 dimensions was classified by the sum of its items in the four categories present in the PHCC, according to an adaptation of Hartz^24^: “adequate” when the presence of 80–100% of items in the PHCC was verified, “partially adequate” when the presence of 60–79% of items was verified, “less adequate” when the presence of 40–59% of items was verified and “inadequate” when the presence of less than 40% of items was verified. Later, the number and percentage of the general structure of essential and strategic dimensions were considered dichotomously as adequate/partially adequate and less adequate/inadequate.

## Measures

Study variables are described in Figure. Explanatory variables were organized in a hierarchical model. The hierarchical analysis model was based on a theoretical model of factors associated with PHCC, in which the hierarchical relationship between the variables associated with under-5 PCSC hospitalizations was considered. Sociodemographic variables are at the farthest level, health care delivery variables are at the intermediary level, and PHCC variable are at the most closest level.

Data corresponding to the PHCC were aggregated at the municipalities level. Socioeconomic and health care delivery variables were already available at the municipalities level, for the ecological hierarchical analysis. Descriptive analyses were conducted with absolute, relative, and mean frequencies and rates. The Kolmogorov-Smirnov test, graphical analyses (box plot and histograms), and an assessment of kurtosis and coefficient of symmetry were used to evaluate the normality of the distribution of under-5 PCSC hospitalization rates. As exploratory analyses showed an asymmetric distribution, nonparametric tests were selected. Based on hierarchical structure, the variables of interest were adjusted by univariate and multivariate negative binomial regression models.

The measure of relative association was the incidence risk ratio (IRR), with respective 95% confidence intervals (95%CI). Variables at the farthest level in the theoretical model were initially included. Variables with p < 0.10 were chosen to remain in the model, after adjustment for variables at the same level. Then, variables at the intermediate level were added to the model, and those with p < 0.10 were selected to remain, after adjustment for variables at the same and previous level. This procedure was repeated until reaching the final model. A level of p < 0.05 was used as the criterion for statistical significance. Analyses were carried out with STATA, version 11.0.

## RESULTS

### Sociodemographic and health care delivery characteristics

Sociodemographic and health care delivery characteristics varied among the Brazilian federative units capitals (Table 1). Capitals in the North and Northeast regions exhibited greater rates of illiteracy than those in the South and Southeast. Illiteracy rates ranged between 1.9% in Florianópolis, Santa Catarina (SC—South Region), and 11.3% in Maceió, Alagoas (AL—Northeast Region). The percentage of the population with access to clean water and sewage systems was also diparate between region, showing higher access in the South and Southeast and lower access in the North and Northeast Regions. Clean water access ranged between 37.7% in Porto Velho, Rondônia (RO—North Region), and 99.7% in Belo Horizonte, Minas Gerais (MG—Southeast Region); the percentage of the population with access to sewage systems ranged between 26.7% in Macapá, Amapá (AP—North Region), and 98.1% in Vitória, Espírito Santo (ES—Southeast Region). The Northeast Region presents the capitals with the lowest and highest FHS coverage, Salvador, Bahia (BA), at 13.3%, and Teresina, Piauí (PI), at 96.5%, respectively.

**Table 1:** Sociodemographic and health services characteristics in the capitals of Brazilian federative units. Brazil, 2012 to 2013.

Capitals (FU)	Demographic density	< 05 years old	Urbanization rate	Illiteracy rate	GDP per capita	Population with water access	Population with sanitation systems	Family health strategy coverage	Public hospital beds

	(inhabitants/km^2^)	(%)	(%)	(% >15 years)	(R$)	(%)	(%)	(%)	(1 000/ inhabitants)
Porto Velho (RO)	12.6	8.3	91.2	5.2	21,784.8	37.7	42.8	58.6	2.2
Rio Branco (AC)	38.0	9.0	91.8	8.9	13,120.2	52.7	56.7	52.4	2.1
Manaus (AM)	158.1	9.0	99.5	3.9	27,845.7	76.0	62.4	27.7	1.6
Boa Vista (RO)	50.0	9.5	97.7	5.7	17,552.7	96.0	54.1	47.5	1.9
Belém (PA)	1 315.3	7.1	99.1	3.3	14,027.1	76.4	67.9	15.8	1.7
Macapá (AP)	62.1	9.8	95.7	6.0	13,821.9	55.7	26.8	45.8	1.5
Palmas (TO)	102.9	8.9	97.1	3.7	15,878.9	95.2	67.6	66.0	1.3

**North**	**248.4**	**8.8**	**96.0**	**5.3**	**17,718.8**	**70.0**	**54.0**	**44.8**	**1.8**

São Luís (MA)	1 215.7	7.4	94.5	4.6	20,242.7	76.6	65.4	27.5	3.0
Teresina (PI)	584.9	7.3	94.3	8.8	13,866.8	93.5	61.6	96.5	2.7
Fortaleza (CE)	7 786.4	6.9	100.0	6.8	16,962.9	93.4	74.0	33.0	2.3
Natal (RN)	4 805.2	6.6	100.0	7.9	15,129.3	98.4	61.8	23.8	2.5
João Pessoa (PB)	3 421.3	7.0	99.6	7.7	13,786.4	78.4	41.6	80.0	3.2
Recife (PE)	7 039.6	6.3	100.0	6.9	21,434.9	87.3	69.2	52.9	4.0
Maceió (AL)	1 854.1	7.7	99.9	11.3	14,572.4	74.3	47.1	30.7	2.6
Aracaju (SE)	3 140.7	7.1	100.0	6.6	15,913.4	97.9	87.2	81.0	2.9
Salvador (BA)	3 859.4	6.2	100.0	3.9	14,411.7	98.9	92.8	13.3	2.0

**Northeast**	**3 745.3**	**6.9**	**98.7**	**7.2**	**16,257.8**	**88.7**	**66.7**	**48.8**	**2.8**

Belo Horizonte (MG)	7 167.0	5.6	100.0	2.8	23,053.1	99.7	96.2	72.6	2.3
Vitória (ES)	3 338.3	6.0	100.0	2.5	85,794.3	99.3	98.1	77.2	3.9
Rio de Janeiro (RJ)	5 265.8	5.8	100.0	2.8	32,940.2	98.3	94.4	39.8	2.0
São Paulo (SP)	7 398.3	6.3	99.1	3.1	42,152.8	99.0	92.6	33.2	1.3

**Southeast**	**5 792.3**	**5.9**	**99.8**	**2.8**	**45,985.1**	**99.1**	**95.3**	**55.7**	**2.4**

Curitiba (PR)	4 027.0	6.2	100.0	2.1	32,916.4	99.2	96.3	36.2	1.9
Florianópolis (SC)	623.7	5.4	96.2	1.9	26,749.3	93.2	87.8	90.4	2.7
Porto Alegre (RS)	2 837.5	5.6	100.0	2.2	32,203.1	99.3	93.0	31.5	3.2

**South**	**2 496.1**	**5.7**	**98.7**	**2.1**	**30,622.9**	**97.2**	**92.4**	**52.7**	**2.6**

Campo Grande (MS)	97.2	7.2	98.7	3.8	19,745.4	90.4	58.7	36.4	1.8
Cuiabá (MT)	157.7	7.4	98.1	4.5	22,301.8	94.0	80.2	38.5	2.0
Goiânia (GO)	1 776.7	6.5	99.6	3.1	20,990.2	92.5	76.1	46.6	2.5
Brasília (DF)	444.7	7.4	96.6	3.6	63,020.0	94.8	87.9	17.6	1.7

**Midwest**	**619.1**	**7.1**	**98.3**	**3.7**	**31,514.4**	**92.9**	**75.7**	**34.8**	**2.0**

Source: IBGE / BRAZIL (2010); SIAB/ DATASUS / BRAZIL

### PHCC structural and organizational characteristics

The percentage of PHCC with an adequate general structure (structural and organizational) for pediatric health care was only 18.9% in the Brazilian capitals. Curitiba, Paraná (PR), and São Paulo, São Paulo (SP), in the South and Southeast regions, respectively, presented the highest proportions of adequate PHCC. Whereas Manaus, Amazonas (AM), and Belém, Pará (PA) in the North, and Maceió, AL, João Pessoa, Paraíba (PB) and Salvador, BA in the Northeast, and Goiânia, Goiás (GO), in the Midwest presented the lowest proportions of adequate PHCC (Table 2). Of all dimensions, hours of operation and instruments presented the highest percentage of adequacy (above 80%), while medications and accessibility presented the lowest proportions of adequacy (respectively below 69% and 42%). A total of 20 of the 27 capitals (74.1%) had PHCC fewer than 10% with adequate general structure. The highest proportions of adequate PHCC were found in the Southeast region (53.9%) and in the capitals of Rio de Janeiro, Rio de Janeiro (RJ—68.2%), São Paulo, SP (65.7%), Florianópolis, SC (63.3%), and Curitiba, PR (67.3%) (Table 2).

**Table 2:** Adequacy of current services in pediatric care in PHC in the capitals of Brazilian federative units. Brazil, 2012 to 2013.

Capitals (FU)	Dimensions of PHC (%)															GENERAL STRUCTURE

	Hours of operation	Recommended no. of professionals per team	No. physicians per team	Equipment	Inputs	Forms	Medications	Vaccines	ESSENTIALS	Environments	Ambience	Structural conditions	Signaling/identification	Accessibility	ESTRATEGIC	
Porto Velho (RO)	97.7	85.4	53.5	22.7	88.6	61.4	0.0	47.7	29.5	6.8	27.3	54.6	4.6	0.0	6.8	6.8
Rio Branco (AC)	94.0	55.0	34.9	20.9	82.1	34.3	9.0	46.3	23.9	16.4	6.0	56.7	4.5	7.5	7.5	7.5
Manaus (AM)	98.2	46.6	29.7	13.3	56.4	24.4	0.0	32.0	15.1	5.8	7.1	51.6	7.6	3.6	4.0	1.3
Boa Vista (RR)	97.2	53.1	45.4	0.0	44.4	52.8	2.8	58.3	25.0	5.6	13.9	44.4	8.3	0.0	2.8	2.8
Belém (PA)	90.4	70.6	29.8	1.4	34.3	39.7	0.0	41.1	2.7	5.5	15.1	52.1	1.4	1.4	2.7	0.0
Macapá (AP)	87.2	80.9	60.5	4.1	26.5	2.0	0.0	38.8	2.0	8.2	10.2	22.5	0.0	2.0	2.0	2.0
Palmas (TO)	96.9	23.3	23.3	18.2	100.0	81.8	0.0	51.5	48.5	3.0	18.2	81.8	21.2	0.0	3.0	6.1
**North**	**94.5**	**59.3**	**35.7**	**11.5**	**61.8**	**42.3**	**1.7**	**45.1**	**17.3**	**7.3**	**14.0**	**52.0**	**6.8**	**2.1**	**4.2**	**2.8**
São Luís (MA)	98.0	67.4	67.4	14.0	68.0	60.0	0.0	60.0	36.0	18.0	32.0	58.0	12.0	4.0	12.0	6.0
Teresina (PI)	80.2	45.1	40.2	24.4	94.9	56.1	0.0	24.4	13.4	4.9	11.0	64.6	2.4	1.2	4.9	2.4
Fortaleza (CE)	100.0	87.9	85.7	37.4	63.7	58.2	1.1	55.0	36.3	25.3	15.4	23.1	5.5	1.1	3.3	3.3
Natal (RN)	100.0	89.4	71.7	17.0	32.1	30.2	0.0	39.6	9.4	15.1	13.2	54.7	1.9	9.4	7.5	3.8
João Pessoa (PB)	98.7	25.7	20.0	12.6	86.8	72.2	0.0	4.0	9.9	12.6	13.9	40.4	1.3	0.0	4.6	1.3
Recife (PE)	99.2	35.9	24.8	22.3	83.5	66.2	12.2	44.6	31.6	5.8	18.7	49.6	25.9	2.2	4.3	2.9
Maceió (AL)	100.0	13.5	10.8	5.4	18.9	35.1	0.0	40.5	2.7	8.1	2.7	32.4	10.8	0.0	0.0	0.0
Aracaju (SE)	100.0	18.6	11.6	65.1	93.0	53.5	37.2	62.8	58.1	27.9	18.6	51.2	11.6	7.0	2.3	7.0
Salvador (BA)	99.0	79.3	61.6	39.3	69.2	55.1	0.0	70.1	31.8	15.9	15.0	24.3	21.5	1.9	6.5	0.9
**Northeast**	**97.2**	**51.4**	**42.1**	**26.4**	**67.8**	**54.1**	**5.6**	**44.6**	**24.7**	**14.8**	**15.6**	**44.3**	**10.3**	**3.0**	**5.0**	**2.7**
Belo Horizonte (MG)	85.5	58.2	41.1	2.7	95.9	64.0	27.9	89.1	61.9	14.3	44.9	76.2	35.4	40.8	42.2	36.7
Vitória (ES)	100.0	80.0	53.9	88.5	100.0	80.8	53.9	50.0	84.6	50.0	42.3	61.5	26.9	30.8	38.5	53.8
Rio de Janeiro (RJ)	98.8	66.9	54.0	63.7	96.1	57.0	68.2	59.8	63.7	41.3	21.8	83.8	41.9	17.3	32.4	38.0
São Paulo (SP)	98.6	69.1	38.8	40.1	94.9	50.1	65.7	84.3	66.5	62.1	34.7	73.3	70.5	20.4	54.1	52.2
**Southeast**	**95.7**	**68.6**	**43.3**	**48.8**	**96.7**	**63.0**	**53.9**	**70.8**	**65.6**	**41.9**	**35.9**	**73.7**	**43.7**	**27.3**	**46.3**	**46.1**
Curitiba (PR)	100.0	58.5	35.0	46.5	98.0	82.2	23.8	95.1	77.2	72.3	59.4	80.2	67.3	20.8	70.3	67.3
Florianópolis (SC)	100.0	83.3	66.7	77.6	100.0	73.5	63.3	36.7	59.2	34.7	34.7	73.5	71.4	12.2	44.9	40.8
Porto Alegre (RS)	99.5	67.8	17.0	35.4	97.1	14.6	7.3	74.3	20.9	11.7	16.5	51.0	10.2	2.9	5.8	3.9
**South**	**99.8**	**69.9**	**28.8**	**53.2**	**98.4**	**56.8**	**31.4**	**68.7**	**42.1**	**39.6**	**36.9**	**68.2**	**49.6**	**12.0**	**29.5**	**27.0**
Campo Grande (MS)	98.3	47.1	38.2	91.4	91.4	91.4	51.7	82.8	91.4	60.3	15.5	69.0	15.5	3.5	17.2	39.7
Cuiabá (MT)	100.0	39.6	20.7	17.2	71.9	54.7	0.0	53.1	28.1	15.6	17.2	50.0	4.7	3.1	9.4	4.7
Goiânia (GO)	97.6	91.5	62.5	10.7	59.5	33.3	0.0	21.4	7.1	15.5	4.8	45.2	13.1	2.4	4.8	2.4
Brasília (DF)	100.0	60.7	13.1	13.8	53.1	53.1	0.0	51.0	12.4	51.0	17.9	53.8	9.7	1.4	15.9	3.4
**Central-west**	**99.0**	**59.7**	**29.8**	**33.3**	**69.0**	**58.1**	**12.9**	**52.1**	**27.1**	**35.6**	**13.9**	**54.5**	**10.8**	**2.6**	**12.2**	**9.4**
**BRAZIL**	**96.9**	**59.3**	**38.0**	**29.8**	**74.1**	**53.2**	**15.7**	**52.4**	**37.3**	**22.7**	**20.3**	**54.8**	**18.8**	**7.3**	**20.6**	**18.9**

Sources: PMAQ-AB (2012).

### Under-5 PCSC hospitalizations

Overall rates of under-5-hospitalizations (per 1,000 inhabitants) were the highest among the most populous capitals, especially in the Southeast region. São Paulo, SP, Rio de Janeiro, RJ, and Belo Horizonte, MG, had the highest rates of 563.2, 197.8, and 123.6 per 1,000 inhabitants, respectively. However, rates of hospitalizations for the three PCSC conditions responsible for the greatest burden of under-5 admissions were the highest in Belem, PA (74.7/1,000), and João Pessoa, PB (53.7/1,000). Florianópolis, SC, presented the lowest of rate hospitalization for the three most prevalent conditions with 8.6/1,000 inhabitants. Pneumonia presents the highest number of hospitalizations (68%), followed by asthma (18%) and diarrhea (14%) (Table 3).

**Table 3:** PCSC hospitalizations in under-5 populations in the capitals of Brazilian federative units. Brazil, 2012 to 2013.

Capitals (FU)	Total	PCSC hospitalizations							

		Pneumonia		Asthma		Diarrhea		Asthma / Diarrhea / Pneumonia	
				
	Rate^a^	n	Rate^a^	n	Rate^a^	n	Rate^a^	n	Rate^a^
**North**									
Porto Velho (RO)	20.76	574	15.71	60	1.64	204	5.58	838	22.94
Rio Branco (AC)	22.57	293	9.30	8	0.25	80	2.54	381	12.09
Manaus (AM)	10.89	4,970	29.59	386	2.30	1,525	9.08	6,881	40.98
Boa Vista (RR)	22.53	836	29.66	58	2.06	229	8.12	1,123	39.84
Belém (PA)	75.13	2,787	27.67	1,942	19.28	2,790	27.70	7,519	74.66
Macapá (AP)	23.74	1,244	30.39	82	2.00	288	7.04	1,614	39.43
Palmas (TO)	16.51	388	18.04	22	1.02	133	6.18	543	25.25
**Northeast**									
São Luís (MA)	55.46	1,236	15.98	52	0.67	450	5.82	1,738	22.47
Teresina (PI)	50.25	296	4.91	141	2.34	169	2.80	606	10.05
Fortaleza (CE)	130.40	2,307	13.40	2,001	11.62	92	0.53	4,400	25.56
Natal (RN)	36.07	699	12.97	193	3.58	44	0.82	936	17.37
João Pessoa (PB)	46.32	2,537	49.15	136	2.63	101	1.95	2,774	53.74
Recife (PE)	97.88	1,502	15.33	816	8.33	285	2.91	2,603	26.58
Maceió (AL)	47.32	2,349	32.19	137	1.88	325	4.45	2,811	38.53
Aracaju (SE)	23.34	508	12.14	400	9.56	172	4.11	1,080	25.80
Salvador (BA)	142.53	2,835	16.93	384	2.29	283	1.69	3,502	20.91
**Southeast**									
Belo Horizonte (MG)	123.56	1,682	12.52	1,563	11.63	243	1.81	3,488	25.96
Vitória (ES)	15.61	273	13.71	119	5.97	6	0.30	398	19.98
Rio de Janeiro (RJ)	197.85	2,880	7.82	223	0.60	293	0.79	3,396	9.23
São Paulo (SP)	563.19	12,751	17.74	2,221	3.09	1,440	2.00	16,412	22.83
**South**									
Curitiba (PR)	99.81	754	6.89	199	1.82	222	2.03	1,175	10.73
Florianópolis (SC)	19.01	163	6.94	27	1.15	12	0.51	202	8.60
Porto Alegre (RS)	96.70	769	9.73	997	12.61	103	1.30	1,869	23.65
**Central-west**									
Campo Grande (MS)	45.70	950	16.29	11	0.19	48	0.82	1,009	17.30
Cuiabá (MT)	31.60	443	10.72	32	0.77	46	1.11	521	12.61
Goiânia (GO)	82.63	2,533	29.27	438	5.06	410	4.74	3,381	39.07
Brasília (DF)	147.76	2,880	14.78	745	3.82	807	4.14	4,432	22.74

^a^ Per 1,000 inhabitants. Source: SIH/DATASUS/BRAZIL (2012).

### Association between Under-5 PCSC hospitalization and PHCC structural and organizational characteristics

The proportion of under-5 population and rate of urbanization (p < 0.10), as covariates at the farthest level, were selected to standardize the models. No variables from the intermediate level were selected for the multivariate model. After adjustment, the characteristics of primary care associated with lower rates of under-5 hospitalizations for the three leading PCSC were considered as the level of adequacy of the following dimensions: equipment (IRR = 0.98; 95%CI 0.97–0.99), structural conditions (IRR = 0.98; 95%CI 0.97–0.99), and signage/identification (IRR = 0.98; 95%CI 0.97–0.99). On the other hand, characteristics associated with the higher rates of hospitalizations were considered as the levels of adequacy of the following dimensions: number of physicians per team (IRR = 1.23; 95%CI 1.02–1.48), availability of forms (IRR = 1.01; 95%CI 1.01–1.02), and medications (IRR = 1.02; 95%CI 1.01–1.03), seen in Table 4. Considering all the aggregated general structure indicators, we found an association between hospitalizations (IRR = 0.99; 95%CI 0.98–0.99) and the variable of general structure, which were inversely associated (Table 4).

**Table 4:** Association between municipality PHCC with pediatric hospitalizations for PCSC in the capital of Brazilian federative units. Brazil, 2012 to 2013.

Variable	Pediatric hospitalizations for PCSC					

	Unadjusted			Adjusted^a^		
	
	IRR	95%CI	P	IRR	95%CI	P
**Farthest level**						
Demographic density	1.00	0.99–1.01	0.110			
% under-5	1.32	1.12–1.55	0.001	**1.29**	**1.14–1.47**	**< 0.001**
Urbanization rate	1.02	0.93–1.12	0.610	**1.07**	**1.02–1.13**	**0.008**
Illiteracy rate	1.05	0.95–1.16	0.310			
GDP per capita	1.00	0.99–1.01	0.070			
% population with access to water supply	0.98	0.97–0.99	0.023			
% population with access to sanitation	0.98	0.97–0.99	< 0.001			
**Intermediate level**						
Family Health Strategy coverage	0.99	0.98–1.01	0.231			
No. public hospital beds (per 1000)	0.83	0.62–1.12	0.220			
**Closest level**						
Essential dimension	0.99	0.98–0.99	< 0.001	0.99	0.98–0.99	0.001
Strategic dimension	0.99	0.98–0.99	< 0.001	0.99	0.98–0.99	0.018
General structure	0.98	0.97–0.99	< 0.001	0.99	0.98–0.99	0.006
Hours of operation	0.97	0.92–1.02	0.275			
Recommended no. of professionals per team	0.99	0.98–1.00	0.433			
Physicians per team	1.08	0.80-1.45	0.610	**1.23**	**1.02–1.48**	**0.027**
Equipment	0.99	0.98–0.99	< 0.001	0.98	0.97–0.99	< 0.001
Inputs	0.98	0.97–0.99	< 0.001			
Forms	0.99	0.98–0.99	0.030	**1.01**	**1.01–1.02**	**0.030**
Medications	0.98	0.97–0.99	< 0.001	**1.02**	**1.01–1.03**	**0.007**
Vaccines	0.98	0.97–0.99	0.009			
Environments	0.98	0.97–0.99	< 0.001			
Ambience	0.97	0.96–0.99	0.001			
Structural conditions	0.98	0.97–0.99	0.001	0.98	0.97–0.99	0.007
Signage/identification	0.98	0.97–0.99	0.001	0.98	0.97–0.99	0.012
Accessibility	0.97	0.95–0.99	0.006			

^a^ Adjusted for covariates: proportion under-5 and urbanization rate.

## DISCUSSION

This ecological study is one of the first to assess the association between adequacy of PHCC, with a hierarchical analysis, and under-5 PSCS hospitalizations. Overall, a low level of adequacy, defined by the essential and strategic elements in PHCC, was found; more than half of the PHCC in the capitals presented inadequate general structure. Previous studies in Brazil have found similar proportions of inadequacy of PHCC as well as disparity among regions, with 37.3% adequacy in the North, 39.6% in the Northeast, 55.9% in the Southeast, 59.9% in the Midwest, and 60.8% in the South^25^. This prevalence of inadequate infrastructure in PHCC assists in describing a main shortcoming in the current health system, particularly regarding the relationship between structural characteristics, access, and quality^26^.

These low levels of structure adequacy in PHCC pose a fundamental challenge for population health and contribute to an increased burden of avoidable diseases and their complications. In this study, the capitals of the North and Northeast regions presented the greatest rates of under-5 PCSC hospitalizations, which had the greatest proportions of inadequate PHCC, the greatest illiteracy rates, the lowest Gross Domestic Product (GDP) per capita, and the lowest coverage of clean water supply and sanitation. Previous research had pointed to higher rates of hospitalizations for PCSC among populations with lower socioeconomic conditions^18^. This research has equally pointed to higher rates of PCSC among population with the worst socioeconomic conditions, since these conditions affect access to health facilities, impair adherence to treatment, and hinder health care understanding and adherence to healthy habits^27^.

The association between urbanization rates and pediatric hospitalizations could be explained by a social vulnerability of the pediatric population in urban locations. In general, urban agglomerations favor the occurrence of respiratory diseases such as asthma and pneumonia. Besides, another potential explanation for the association between urbanization and pediatric hospitalization lies in the socioeconomic and environmental factor of urbanization^8^. Factor such as an easier access to hospitals by those living in urban centers, such as capitals, could favor a higher hospitalization usage or specialized care as an entry door to the health system^27^.

A key contribution of our study is identifying variables at different levels that are risk factors or protective factors regarding rates of under-5 hospitalizations. The variables number of physicians per team, availability of medication, and forms were significant risk factors.

This result seems to be unexpected, as greater adequacy in these dimensions are associated with a reduction in hospitalizations^28^. However, considering the structure of the Brazilian health care system, it is possible that well-resourced PHCC are also more likely to refer pediatric patients to more specialized levels of care considering they are located in an area where this option is possible. Thus, the association observed in our study is similar to moral hazard theories in health care, which suggest that the use and accessibility of health facilities are positively related. Note that this study was conducted in state capitals only. Thus, the result possibly suggesting higher number of physicians and medications was associated with higher under-5-hospitalization. This association could be a proxy to the biomedical model of care in such locations. This result is not coherent with the rationale of the FHS model, which are more present in less developed areas where higher equity in care is necessary. It is known that health teams that are not physician-centered have better performance in health promotion, disease prevention, integrality in health and longitudinal follow up in health care^29^.

The equipment, structural conditions, and signaling/identification variables were considered as protective factors. The availability of these components was directly related to the adequacy of primary care infrastructure, which was related to lower rates of under-5-hospitalizations for PCSC. This relationship could be explained by the fact that quality of health facilities is a function of equipment and structural conditions.

The availability of equipment facilitates prevention and early diagnosis of disease, meeting the pediatric needs at the first level of care without complication, for example. Items such as stethoscope and lantern help in physical examination and diagnostic procedures, and they highlight the importance of clinics in patient care in a basic health unit (BHU). Similarly, the vaccine room is essential to maintain the quality of immunobiological materials, to help in the prevention of diseases and consequently reducing hospitalizations. However, characterizing the association between elements of structure and rate of hospitalization for PCSC underscores the role of structure conditions as both a component of primary care quality and an agent for improving individual and population health^30^. This link is additionally supported by the association between the highest inadequacy of primary care infrastructure in some capitals and their highest rates of under-5-hospitalizations for PCSC.

It is important to highlight the limitations of this study, such as the use of secondary data, which can generate biases due to underreporting. We struggle to minimize these effects by collecting data on hospitalizations only in the capitals and working with official data from the government information systems. One strength of this study is the fact that data collection was performed in a health care-based study by a census of all Brazilian primary care centers using one instrument for the entire national territory.

The findings of this study may contribute to a better reflection on the unnecessary pediatric hospitalizations due to primary care sensitive conditions in the capitals of Brazil, which generate high costs to the health system. This study shows that efforts to improve the physical infra-structure of BHU are essential to enhance the primary care and to reduce the unnecessary hospitalizations burden to the health system as well as to prepare and to organize the advance of urban growth challenges and the demand of health care professionals education. These reflections should be considered when discussing changes in the national policy of primary, child, and maternal health care.
